# Training load and injury surveillance in Leinster SChoolboy RUgby players: the SCRUm cohort study

**DOI:** 10.1136/bmjsem-2025-002535

**Published:** 2025-08-28

**Authors:** Sarah J Murphy, Louise Keating, Ronan Conroy, Jennifer Murphy, Chloe Leddy, David Clancy, Cliff Beirne, John F Quinlan, Chris Bleakley, Helen P French

**Affiliations:** 1 School of Physiotherapy, RCSI University of Medicine and Health Sciences, Dublin, Ireland; 2 Division of Population Health Sciences, Royal College of Surgeons in Ireland, Dublin, Ireland; 3 Irish Rugby Football Union Charitable Trust, Dublin, Ireland; 4 Sport and Exercise Science Research Institute, University of Ulster, Newtownabbey, UK

**Keywords:** Rugby, Training, Athlete, Adolescent, Sporting injuries

## Abstract

**Objectives:**

To describe training loads and injury incidences, and explore their relationship in senior schoolboy rugby players in Leinster, Ireland.

**Methods:**

Prospective cohort study conducted during the 2019–2020 season. Methods aligned with consensus statements for rugby injury surveillance research. Injuries were coded using the Orchard Sports Injury Classification System (OSICS) version 10.1 and recorded using the World Rugby Injury Surveillance System. Training load measures (session type, duration and rate of perceived exertion) were recorded by the players using The Sports Office smartphone application.

**Results:**

In total, 463 participants (mean age 17, (SD=0.9 years)) in 16 schools provided data over 20 weeks. Injury incidence per 1000 player hours was 19.9 (95% CI: 15.2 to 25.6) match injuries and 0.7 (95% CI: 0.4 to 1.0) training injuries. Median injury severity was 22 days (95% CI: 17 to 28) lost for match injuries and 14 days (95% CI: 5 to 41) lost for training injuries. Frequent injury sites included shoulder (n=23, 27%), head (n=22, 26%), wrist/hand (n=9, 11%), ankle (n=8, 10%) and knee (n=5, 6%). Tackle accounted for 49% of injuries. Players’ recording of individual training load showed 11.5% compliance. Exposure was calculated as 31 141 training hours and 3063 match hours. Training sessions included conditioning weights (31%), rugby skills: non-contact (28%) and semicontact (24%), with variation in proportion of sessions across schools.

**Conclusion:**

Match activity and tackle events accounted for most injuries. Training exposure and type varied widely across schools. Low compliance in recorded individual training load limited analysis of association with injury risk, highlighting the challenge in identifying injury risk factors in an adolescent cohort.

**WHAT IS ALREADY KNOWN ON THIS TOPIC**

- Match injury incidence in schoolboy rugby players varies internationally, with limited data on training injury incidence, training load and practices.

**WHAT THIS STUDY ADDS**

- This prospective cohort study describes both match and training injury incidence in the Leinster schoolboy cohort with match injury incidence of 19.9 injuries (95% CI: 15.2 to 25.6) per 1000 hours and training injury incidence of 0.7 injuries (95% CI: 0.4 to 1.0) per 1000 hours.
- The tackle accounted for 49% of injuries, with the tackler getting injured more frequently (30%) than the ball carrier (19%).
- Training practices varied widely across schools in terms of proportion of training types undertaken, with non-contact rugby skills most frequently reported. Low player compliance in recording individual training load poses a challenge in determining its association with injury risk.

**HOW THIS STUDY MIGHT AFFECT RESEARCH, PRACTICE OR POLICY**

- This study added to the evidence regarding injury incidence in adolescent rugby and allows comparison with other injury incidence studies internationally in this cohort.
- Findings can inform injury prevention practices.
- Results identify challenges in recording training load in this cohort.

## Background

 Rugby union is a field-based contact team sport demonstrating worldwide growth^1^ and increased participation across all age groups.^2 3^ In 2022, 16% of male adolescent respondents reported rugby participation at secondary school and community levels.^4^ Rugby involves physical contact and collisions, and its inherent risk is mitigated by ensuring adherence to the laws of the game, player preparedness and safe conduct.^5^ Age-grade variations of standard laws are in place for players aged under 19 years, supporting graded exposure to contact components of the game over their early playing years.^4^,^6^

Multiple studies have established the incidence and nature of injuries sustained in male adolescent rugby players and mostly for matches.^7^,^10^ A recent meta-analysis reported a pooled match injury incidence of 39.8 injuries/1000 match hours (95% CI: 10.2 to 69.3) in male adolescent rugby players (aged 15–18 years).^10^ Prospective injury surveillance studies in premier level of schools rugby competition in Irish provinces have reported match injury incidences per 1000 match hours of 29.1 in Ulster^11^ and 53.6 in Connacht/Munster.^12^ This shows differences in injury incidences across different regions, at the highest level of schools rugby competition.

Training load measures specific to the player (internal measures), such as heart rate and rate of perceived exertion (RPE), are used along with external measures, such as training duration and type, to monitor players’ response to training.^13 14^ The relationship between training load and injury risk has been explored in many sports.^13^ The arbitrary unit (AU) of training load, using the product of the session RPE and training duration, is a metric of athletes’ response to training, using a ratio of acute workloads over chronic workloads (ACWR).^15 16^ Previous studies in adult team sports have shown a strong non-linear relationship between ACWR and injury risk.^15^ An optimal ACWR range of 0.8–1.5, referred to as the ‘sweet spot’, was associated with reduced injury risk, with an ACWR >1.5 associated with higher risk of injury.^15 16^ Although no published studies to date have explored the association between ACWR and injury risk in adolescent rugby players, an Australian study explored the match and training volumes of 90 adolescent rugby players.^17^ Based on training and match minutes (volume), collected across a median of 11 weeks, a 41% increased odds (OR 1.41; 95% CI: 1.14 to 1.74) of injury was reported, with higher match volumes in weeks preceding injury.^17^

To date, no prospective study has reported match or training injury incidence in Leinster senior schoolboy rugby players or explored training loads and practices, along with individual injury risk factors. Therefore, this study aimed to (1) describe the injury incidence, mechanism, site, nature and severity of injuries, (2) monitor training loads in Leinster Schools’ Senior Cup squads and (3) explore the relationship between training load and injury risk.

## Methods

A prospective cohort study was undertaken in Leinster senior schoolboy rugby squads during the 2019/20 rugby season, with ethical approval from the Royal College of Surgeons in Ireland (RCSI) Research Ethics Committee (REC-001607).

Leinster Rugby Schools Committee members provided advice and support in relation to outcome measures of importance and recruitment of schools to the study. Due to time constraints, it was not possible to involve schoolboy players in the study design.

Players were recruited from schools eligible to compete in the premier level of Leinster senior schools’ competitions (Leinster Schools Senior Challenge Cup (Senior Cup) and Vinnie Murray Cup). These schools competed under age-grade regulations, including full contact and playing 35 min per half.^6^ Leinster Schools rugby season can include preseason either prior to or during September, league and friendly matches from September to December, and Cup competitions between January and March. Recruitment commenced from September to November 2019, and 26 eligible schools were invited to participate via meetings and information events. During this period, the players’ electronic consent and assent (with guardian consent for players aged under 18 years) was obtained. After consent/assent was obtained, data collection took place from 31 October 2019 until 13 March 2020. At baseline, study researchers measured height and weight, and players self-reported injury history, rugby and other sports participation history, and personal protective equipment usage at baseline. In season, players recorded individual training load variables (training session type, duration, intensity (RPE)) for their school rugby participation only and recorded type of other sports participation using an athlete monitoring smartphone application (The Sports Office UK Ltd., Wigan, UK) in line with recommendations.^13^ Players were asked to record daily school rugby training load as frequently as daily or 7 days retrospectively, to reduce data collection burden and limit recall bias.^18^

Injury surveillance was concurrently performed by trained injury data collectors, including school coaches (rugby, and strength and conditioning coaches) (n=11), directors of rugby (n=3), physiotherapist (n=1) and nurse (n=1), via World Rugby’s Injury Surveillance System (ISS) web portal. Injury surveillance was informed by the consensus statement for rugby,^19^ and reporting guidelines for cohort studies.^14 20^ Injuries sustained due to rugby participation (match or training) which resulted in time loss (24 hours) from rugby participation were recorded.^19^ Injury site, nature, mechanism, severity (time loss in days), recurrence and activity (match or training) were recorded.^14 19^ Injuries were coded using the Orchard Sports Injury Classification System (OSICS) version 10.1 within the ISS.^21 22^ Injury data collectors were trained by the research team in use of ISS for recording injuries. This training included explanations of injury definitions (24-hour time loss), the OSICS coding system and its different tiers, practical injury data recording and a written guidebook with a copy of all OSICS codes. Injury data collectors were asked to record a minimum of two tiers of OSICS coding, that is, tier one indicating anatomical site and tier two indicating pathology.^21^

Study researchers contacted each school by phone and/or email on a weekly to fortnightly basis and offered to attend in person to assist as required. This allowed researchers to engage with players to promote ongoing data collection of training load over the study period and address queries relating to training load. Players were incentivised with a gift voucher for ‘most engagement’ over the study period. The researchers also provided support to injury data collectors with injury surveillance, including review of the injury data for accuracy.

### Statistical analysis

Frequency counts and proportions were used to present categorical data. Continuous data were tested for normality using Shapiro-Wilk test. Data were reported as means and SD when normally distributed, with non-normally distributed data reported as medians and IQR (or 95% CIs).

Training exposure was estimated at a team level, using players’ reported training load data.^23^ In consultation with schools during recruitment, coaches confirmed mostly consistent training schedules (session types and duration) each week. If players in the same squad provided differing training session types, the most frequently reported training session type was used based on assurances given by coaches. Training exposure was calculated when at least one player per squad provided training session type and duration. The calculation used the mean training duration for each session, multiplied by the number of players in the school squad (assuming all players participated in training). Match exposure was calculated based on 15 on-field players per 70 min match, multiplied by number of matches per school.^19^

Injury incidence and 95% CIs were calculated using a Poisson distribution and reported as the number of injuries per 1000 exposure hours by activity (match or training). Injury severity was reported as median days lost due to injury, with 95% CIs. Injury burden was calculated using mean time loss from injury and injury incidence.^24^

The initial plan for analysing injury risk involved examining the relationship between ACWR and injury risk using multiple regression models. However, only 11.5% of RPE data was reported by players. Due to insufficient training load data, this analysis could not be completed, and training load data were not used in injury risk analysis.

Alternative statistical approaches were subsequently explored. Zero-inflated Poisson (ZIP) regression involving only baseline characteristics was deemed suitable as over-dispersion only arose due to the excess zeros (large numbers of players with no injuries).

Injury risk analysis used non-time-varying covariates (baseline characteristics) to investigate association with injury frequency (number of injuries sustained). Therefore, only players with complete baseline characteristics data were included in the ZIP regression. ZIP regression modelled count data with excessive zero counts (participants with no reported injuries). The ZIP regression included a Poisson count model and a logit model (logistic regression) to predict excess zeros. The ZIP regression used the number of injuries per player (dependent variable), baseline reported covariates (independent variables), including previous injuries (rugby related or non-rugby related), rugby experience, height, weight, and match and training hours for each school (exposure). Univariate ZIP regression models were used to measure the association between the baseline covariates and players’ injury frequency during the study. Incidence rate ratios with 95% CIs were reported. Statistical significance was set at p<0.05. All analyses were conducted using Stata (StataCorp, College Station, TX, USA).

### Equity, diversity, and inclusion statement

The author group is gender balanced and includes junior and senior researchers across multiple disciplines. Our study population only consisted of male adolescent athletes in schools’ rugby as there was no equivalent Leinster senior schools’ competition for female adolescent rugby athletes at the time of the study.

## Results

**Figure 1:**
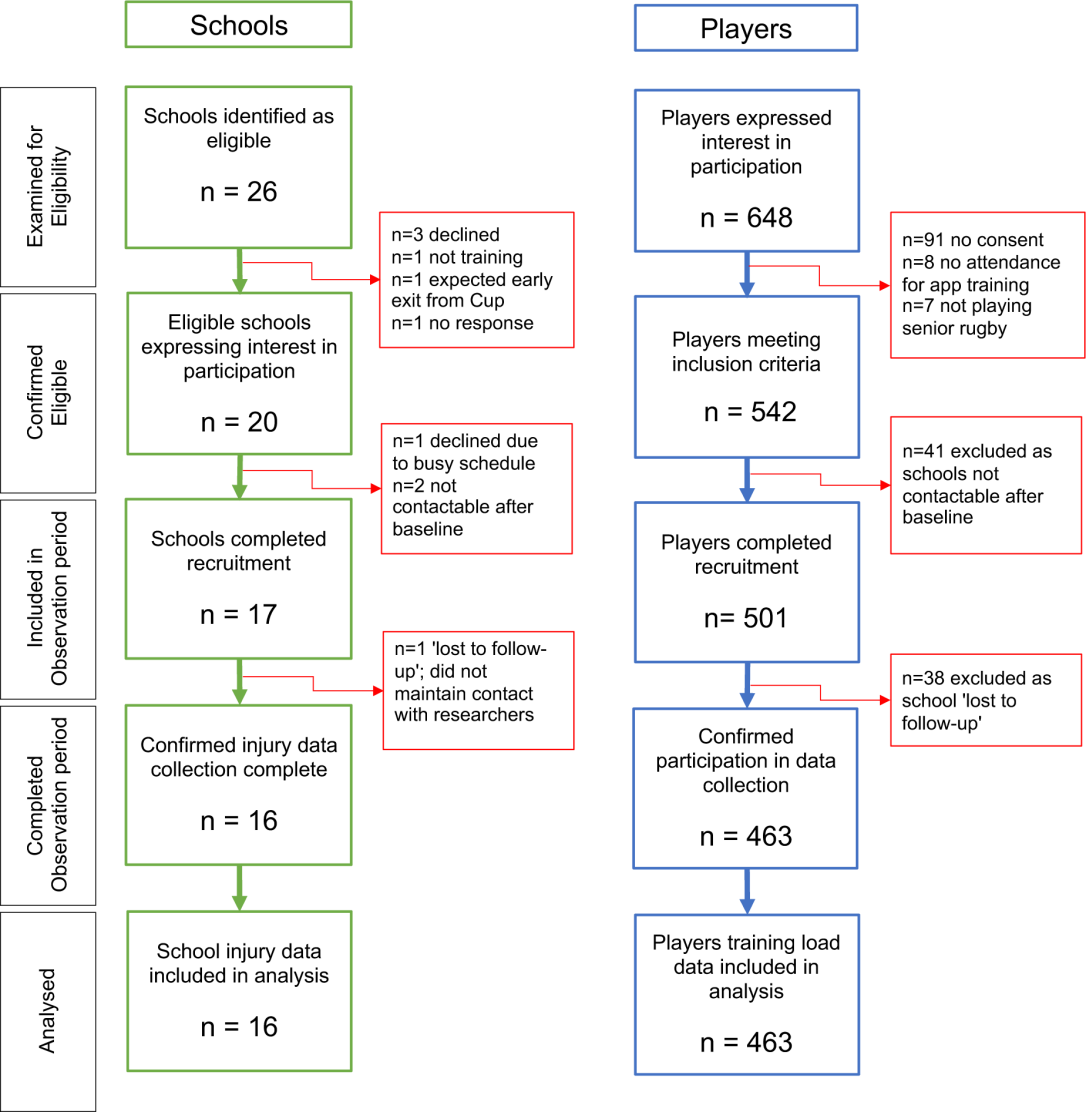
Flow diagram of school and player participation.

A total of 501 participants were recruited from 17 Leinster schools (12 Senior Cup and five Vinnie Murray Cup). One school with 38 participants was lost to follow-up, and results of the remaining 463 participants from 16 schools are reported (figure 1). Players’ baseline characteristics are reported in table 1. Participants’ mean age was 17 (SD=0.9) years, with a median of 10 years (IQR 6, 12) rugby experience, and 32% (n=124) played another sport concurrently with school’s rugby. Injury sustained within the previous 12 months was reported by 54% (n=134) of respondents, and 45% (n=113) reported sustaining more than one injury.

**Table 1:** Participant characteristics

	N (% reported)	Missing (n)	Mean±SD
Participant demographics	463	0	
Age (years)	463 (100)	0	17.0±0.9
Height (cm)	374 (81)	89	181.2±6.2
Weight (kg)*	374 (81)	89	80.7 (74.4, 88.2)
Rugby playing history	276	187	
Age started playing rugby*	276 (100)	187	7 (6, 11)
Years playing rugby*	276 (100)	187	10 (6, 12)
Years of regular training*†	275 (99.6)	187	7 (5, 9)
Playing positions (jersey number)	276	187	
Forwards	152 (55)		
Props (1 or 3)	44 (16)		
Hooker (2)	18 (7)		
Locks (4 or 5)	42 (15)		
Flankers (6 or 7)	37 (13)		
No. 8	11 (4)		
Backs	124 (45)		
Scrum half (9)	22 (8)		
Fly half (10)	21 (8)		
Centres (12 or 13)	30 (11)		
Wingers (11 or 14)	35 (13)		
Full back (15)	16 (6)		
Rugby formats played	277	186	
7s	87 (31)		
10s	29 (10)		
Other	16 (6)		
Personal protective equipment usage	271	192	
Mouth guard	268 (99)		
Head guard	50 (18)		
Shoulder pads	33 (12)		
Other	2 (1)		
Injury history (prev. 12 months)	247	216	
1 injury	134 (54)		
2 injuries	70 (28)		
3+ injuries	43 (17)		
Other sports participation	387	76	
1 sport	124 (32)		
2 sports	116 (30)		
3+ sports	147 (38)		

* Reported as median (IQR) for non-normally distributed data.

† Years regular weight training (two or more days a week).

cm, centimetre; kg, kilogram; n, number.

Data collection was completed over 20 weeks (31 October 2019 until 13 March 2020), with a typical season estimated to be 26–30 weeks (September to March). The COVID-19 pandemic restrictions imposed on 13 March 2020 resulted in a premature end to data collection by approximately 2 weeks. A total of 84 injuries were sustained over 20 weeks, which included 61 (73%) match injuries and 23 (27%) training injuries. Match injury incidence was 19.9 (95% CI: 15.2 to 25.6) per 1000 match hours, with median severity of 22 days (95% CI: 17 to 28). Training injury incidence was 0.7 injuries (95% CI: 0.5 to 1.1) per 1000 training hours, with median severity of 14 days (95% CI: 5 to 41).

Match and training injury incidence by body region, type and severity are presented in tables2  3 respectively. Head injuries (n=20, 33%) were the most frequently reported match injuries. Concussion (n=11, 18%) was the most frequently reported match injury type, with an injury incidence of 3.6 (95% CI: 1.8 to 6.4). Shoulder sprains and subluxations (n=6, 10%) were the second most frequently reported match injuries, with incidence of 2.0 (95% CI: 0.7 to 4.3). In training, shoulder (n=6, 26%) and ankle (n=5, 22%) injuries were most frequent, with a similar incidence of 0.2 (95% CI: 0.1 to 0.4).

**Table 2:** Match injury incidence by region, type and severity

Region (OSICS tier 1)	Injuries	Incidence		Median time loss	
** **Type (OSICS tier 2)	n	per 1000 match hours (95% CI)		Days (95% CI)	
** ** *** **Diagnosis (OSICS tier 3)*					
Total	61	19.9	(15.2 to 25.6)	22	(17 to 28)
Head	20	6.5	(4.0 to 10.1)	24	(14 to 33)
Concussion	11	3.6	(1.8 to 6.4)	29	(18 to 48)
Head laceration/abrasion	5	1.6	(0.5 to 3.8)	14	(4 to 25*)
Head/facial fracture	2	0.7	*(0.1 to 2.4*)	33	(33 to 33*)
* Nasal fracture*	*2*	*0.7*	*(0.1 to 2.4*)	*33*	*(33 to 33* * *)*
Shoulder	17	5.6	(3.2 to 8.9)	22	(14 to 45)
Shoulder sprains/subluxation	6	2.0	(0.7 to 4.3)	40.5	(21 to 53*)
Shoulder soft tissue bruising	3	1.0	(0.2 to 2.9)	23	(18 to 28*)
Shoulder muscle strain	3	1.0	(0.2 to 2.9)	18	(14 to 22*)
Shoulder dislocation	3	1.0	(0.2 to 2.9)	55	(47 to 63*)
Wrist & hand	6	2.0	(0.7 to 4.3)	21	(5 to 61)
Wrist & hand fractures	3	1.0	(0.2 to 2.9)	19	(12 to 63*)
Wrist & hand dislocations	1	0.3	(0 to 1.8)	45	(45 to 45*)
Wrist & hand joint injury	1	0.3	(0 to 1.8)	4	(4 to 4*)
Wrist & hand muscle injury	1	0.3	(0 to 1.8)	22	(22 to 22*)
Knee	4	1.3	(0.4 to 3.3)	44	(22 to 79*)
Knee sprains/ligament Injuries	1	0.3	(0 to 1.8)	Not available†	
Knee cartilage injury	1	0.3	(0 to 1.8)	44	(44 to 44*)
Knee laceration/abrasion	1	0.3	(0 to 1.8)	22	(22 to 22*)
Unknown	*1*	*0.3*	*(0 to 1.8*)	79	(79 to 79*)
Ankle	3	1.0	(0.2 to 2.9)	36	(27 to 59*)
Ankle sprains	3	1.0	(0.2 to 2.9)	43	(27 to 59*)
Thigh	3	1.0	(0.2 to 2.9)	26	(15 to 28*)
*Hamstring strain*	*2*	*0.7*	*(0.1 to 2.4*)	19	(26 to 28*)
Thigh soft tissue bruising	1	0.3	*(0.1 to 2.4*)	15	(15 to 15*)
Chest	3	1.0	(0.2 to 2.9)	13	(8 to 28*)
Hip & groin	1	0.3	(0 to 1.8)	Not available†	
*Hip flexor muscle strain*	*1*	*0.3*	*(0 to 1.8*)	Not available†	
Lumbar spine	1	0.3	(0 to 1.8)	56	(56 to 56*)
Elbow	1	0.3	(0 to 1.8)	Not available†	
Lower leg	1	0.3	(0 to 1.8)	Not available†	
Trunk and abdominal	1	0.3	(0 to 1.8)	2	(2 to 2*)

* Lower (upper) confidence limit held at minimum (maximum) of sample.

† Return to play data not reported, therefore median time loss not available.

OSICS, Orchard Sports Injury Classification System.

**Table 3:** Training injury incidence by region, type and severity

Region (OSICS tier 1)	Injuries	Incidence		Median time loss	
Type (OSICS tier 2)	n	per 1000 training hours (95% CI)		Days (95% CI)	
* * *** *** *Diagnosis (OSICS tier 3)*					
Total	23	0.7	(0.5 to 1.1)	14	(5 to 41)
Shoulder	6	0.2	(0.1 to 0.4)	6	(3 to 58*)
Shoulder sprains/subluxation	2	0.1	(0.0 to 0.2)	58	(58 to 58*)
Shoulder soft tissue bruising	3	0.1	(0.0 to 0.3)	6	(3 to 6*)
*Other soft tissue/bruising*	*1*	*0.0*	*(0.0 to 0.2*)	4	(4 to 4*)
Shoulder muscle strain	1	0.0	(0.0 to 0.2)	7	(7 to 7*)
Ankle	5	0.2	(0.1 to 0.4)	46	(40 to 196*)
*Ankle lateral ligament sprain*	*3*	*0.1*	*(0.0 to 0.3*)	120	(43 to 196*)
*Ankle syndesmosis sprain*	*2*	*0.1*	*(0.0 to 0.2*)	44	(40 to 48*)
Wrist & hand	3	0.1	(0.0 to 0.3)	15	(15 to 15*)
*Fractured thumb*	*1*	*0.0*	*(0.0 to 0.2*)	Not available†	
Wrist & hand dislocations	1	0.0	(0.0 to 0.2)	Not available†	
Wrist & hand soft tissue bruising	1	0.0	(0.0 to 0.2)	15	(15 to 15*)
Head	2	0.1	(0.0 to 0.2)	21	(21 to 21*)
Concussion	2	0.1	(0.0 to 0.2)	21	(21 to 21*)
Thigh	2	0.1	(0.0 to 0.2)	7	(1 to 12*)
*Hamstring strain*	*1*	*0.0*	*(0.0 to 0.2*)	12	(12 to 12*)
*Quadriceps strain*	*1*	*0.0*	*(0.0 to 0.2*)	1	(1 to 1*)
Knee	1	0.0	(0.0 to 0.2)	Not available†	
Knee sprains/ligament injuries	1	0.0	(0.0 to 0.2)	Not available†	
Hip & groin	1	0.0	(0.0 to 0.2)	22	(22 to 22*)
*Hip flexor muscle strain*	*1*	*0.0*	*(0.0 to 0.2*)	11	(22 to 22*)
Lumbar spine	1	0.0	(0.0 to 0.2)	13	(13 to 13*)
Foot	1	0.0	(0.0 to 0.2)	4	(4 to 4*)
Neck	1	0.0	(0.0 to 0.2)	Not available†	

* Lower (upper) confidence limit held at minimum (maximum) of sample.

† Return to play data not reported, therefore median time loss not available.

OSICS, Orchard Sports Injury Classification System.

**Figure 2:**
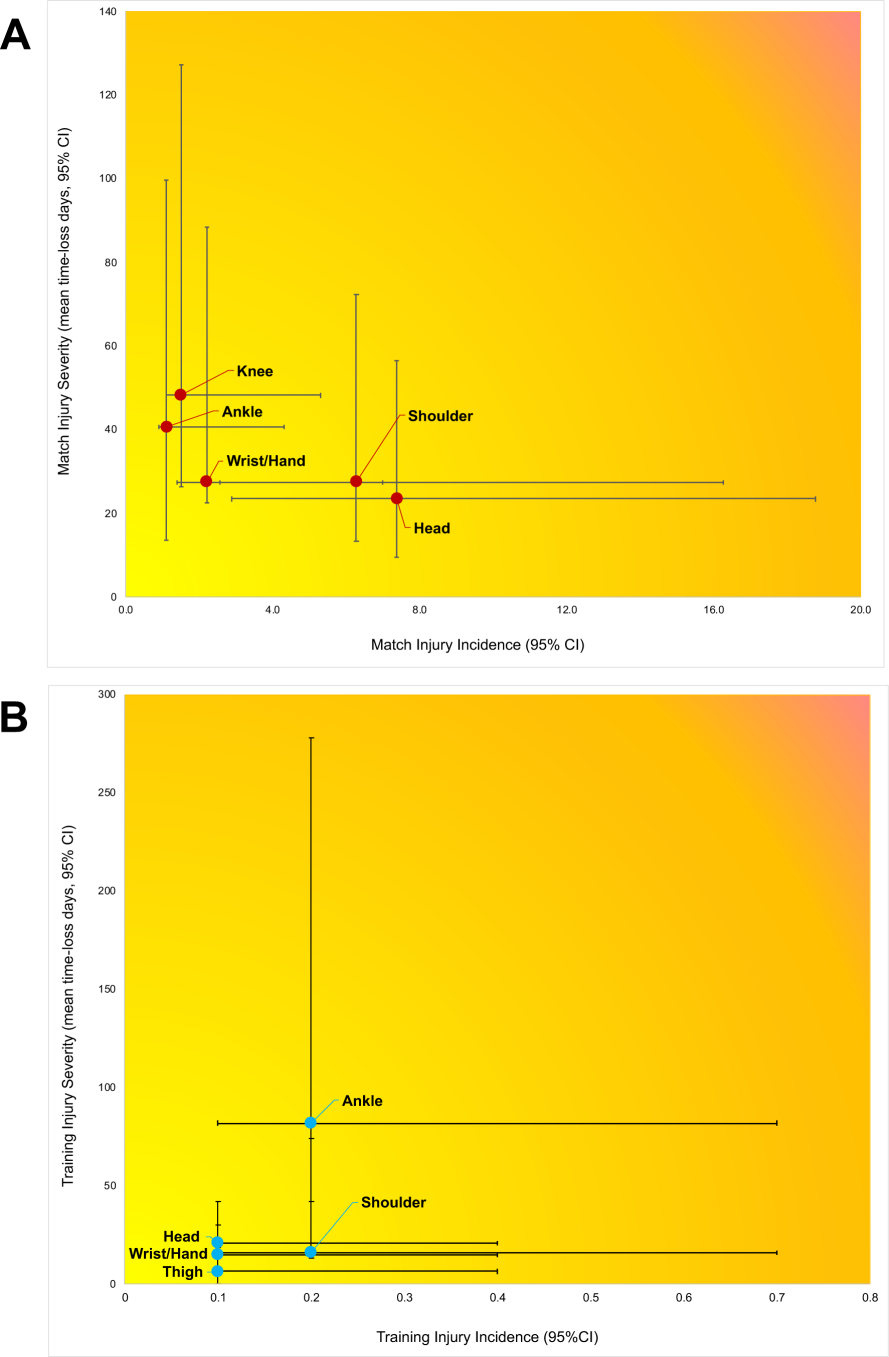
Match (**A**) and training (**B**) injury burden matrices, by most frequent injury regions.

Match injury burden showed a higher incidence of head and shoulder injuries, with a greater time loss for knee and ankle injuries (figure 2A). Training injury burden showed ankle injuries had both a high incidence and time loss compared with other areas and match injuries (figure 2B).

Six injuries were categorised as recurrent or subsequent injuries, with five players sustaining multiple injuries. Recurrent injuries included shoulder injuries (sprains/subluxation; n=1, muscle/soft tissue; n=2). Subsequent injuries included shoulder tendon injury (n=1), knee ligament injury (n=1) and nasal fracture (n=1).

The tackle accounted for 49% of all injuries (table 4), with the tackler more frequently injured (30%) than the ball carrier (19%). In matches, tackling (n=21, 25%), being tackled (n=16, 19%) and collisions (n=11, 13%) incited the majority of injuries. Tackling, collisions, running and rucks incited equal proportions of training injuries (n=4, 5%). Further details of mechanism of injury by activity and site are provided in online supplemental material. Forwards (n=47, 56%) sustained more injuries than backs (n=32, 38%) (see online supplemental material).

**Table 4:** Mechanism of injury, by activity

Match injuries				Training injuries			
Mechanism	N (%)	Incidence per 1000 match hours (95% CI)	Median time loss Days (95% CI)	Mechanism	N (%)	Incidence per 1000 training hours (95% CI)	Median time loss Days (95% CI)
Total	61 (73)	19.9 (17.2 to 28.9)	22 (17 to 28)	Total	23 (27)	0.7 (0.5 to 1.1)	14 (5 to 41.5)
Tackling	21 (34)	6.9 (4.8 to 11.8)	21 (14 to 28)	Tackling	4 (17)	0.1 (0.0 to 0.4)	18 (6 to 43*)
Tackled	16 (26)	5.2 (3.4 to 9.6)	27 (12 to 42)	Tackled	0 (0)	–	–
Collision	11 (18)	3.6 (2.0 to 7.3)	17.5 (5 to 27)	Collision	4 (17)	0.1 (0.0 to 0.4)	7 (7 to 7*)
Running	5 (8)	1.6 (0.6 to 4.3)	36 (26 to 59*)	Running	4 (17)	0.1 (0.0 to 0.4)	17 (1 to 196*)
Ruck	3 (5)	1 (0.2 to 3.2)	57.5 (52 to 63*)	Ruck	4 (17)	0.1 (0.0 to 0.4)	4 (3 to 48*)
Other	3 (5)	1 (0.2 to 3.2)	22 (8 to 32*)	Other	3 (13)	0.1 (0.0 to 0.3)	31 (4 to 58*)
Maul	1 (2)	0.3 (0.0 to 2.1)	–	Maul	1 (4)	0 (0.0 to 0.2)	Not reported
Not known	1 (2)	0.3 (0.0 to 2.1)	–	Not known	1 (4)	0 (0.0 to 0.2)	13 (13 to 13*)
Scrum	0 (0)	–	–	Scrum	1 (4)	0 (0.0 to 0.2)	Not reported
Lineout	0 (0)	–	–	Lineout	1 (4)	0 (0.0 to 0.2)	40 (40 to 40*)

* Lower (upper) confidence limit held at minimum (maximum) of sample.

n, number.

Mean study participation period was 16 (SD=2) weeks across the 20-week observation period. Schools competed in 175 matches, with a mean of 11 (SD=4) matches per school. Total match exposure was 3063 hours.

Schools trained a mean of 3 (SD=1.4) sessions per week and mean of 4 (SD=1) hours weekly. The most frequent training session types were conditioning weights (31%), non-contact rugby skills (28%) and semicontact rugby skills (24%). The proportion of session types undertaken varied widely across schools (see online supplemental material). Total training exposure was estimated at 31 141 hours. It was estimated that 11.5% of RPE (internal training load) data was recorded by players. Consequently, due to 88.5% missing individual training load data, it was not possible to explore associations between training load (ACWR) and injury risk.

**Figure 3:**
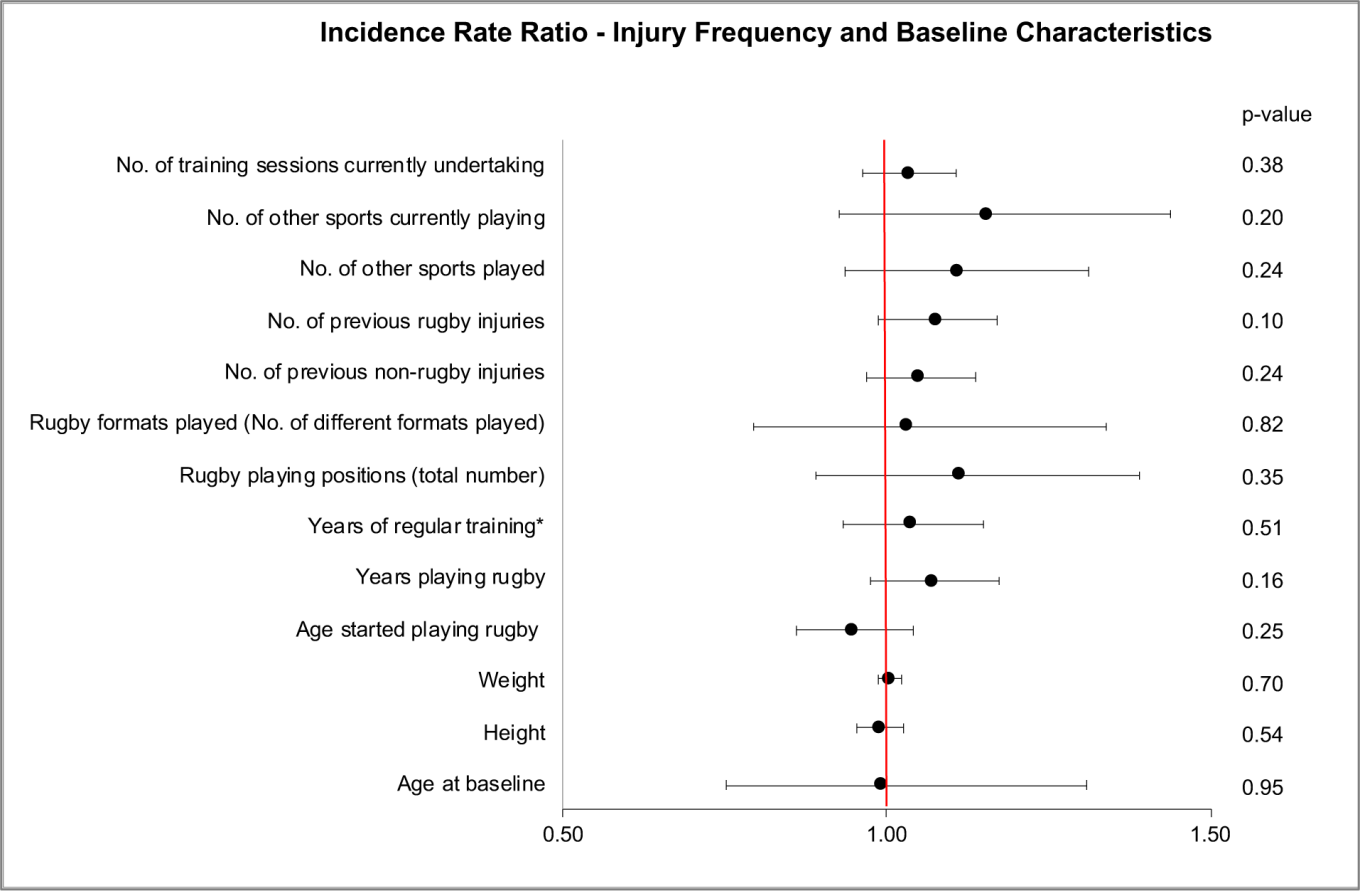
Incidence rate ratio of injuries sustained (frequency) and baseline characteristics. *regular defined as two weight sessions per week.

Injury risk analysis, using a zero-inflated Poisson regression, showed no significant association between number of injuries sustained during the study and baseline characteristics including previous injuries (rugby related or non-rugby related), rugby experience, height and weight (figure 3).

## Discussion

This cohort study reported a lower match injury incidence (per 1000 match hours) than other Irish provincial schoolboy studies in Ulster (29.1) and Munster/Connacht (53.6), and English schoolboy studies (35, 95% CI: 29 to 41).^11 12 25^ Training injury incidence (per 1000 training hours) was also lower compared with English schoolboy studies (2, 95% CI: 2 to 3; 2.1, 95% CI: 1.4 to 2.9).^23 25^ These variations in injury incidences are reported in several systematic reviews of injury surveillance in youth male rugby players.^7 8 10 26^ The most recent meta-analysis reported an overall match injury incidence of 31 (95% CI: 7 to 54) in males aged 15–18 years (range 17.2, 95% CI: 14 to 21 to 138, 95% CI: 137 to 140).^10^ The match injury incidence reported in our study lies within this range. It is difficult to ascertain if factors such as study methodology, competition level, level of experience, governance of play, maturation and other sports participation influence this variation in injury incidence and further research to explore relevant factors is recommended.

A total of 20 weeks of injury surveillance during mid to late season was completed, with approximately 8 weeks of early season not surveyed. Higher proportions of early season injuries (training 73% of injuries, match 47%) were reported in 156 adult semiprofessional rugby league players.^27^ Early season injuries not accounted for in this study could potentially impact the injury incidence reported.

Consistent with other youth studies, a lower injury incidence compared with amateur and elite adult players was found. A meta-analysis of amateur adult male rugby players reported match injury incidence 46.8/1000 hours (95% CI: 34.4 to 59.2).^28^ A meta-analysis of elite male rugby players reported match injury incidence of 91/1000 match hours (95% CI: 77 to 106) and training injury incidence of 2.8/1000 training hours (95% CI: 1.9 to 4.0).^29^ This difference highlights the need to consider adult and youth cohorts independently when addressing injury risk.

The nature of injuries appears similar, with injuries to the head, shoulder, knee, ankle and hand/finger/thumb common across Irish schoolboy studies.^11 12^ While injury risk factors identified in other adolescent studies include younger age, higher playing level, higher weight and height, previous injury, playing position and regular weight training,^11 30 31^ we did not find an association between these risk factors and increased injury risk. The concussion injury incidence of 3.6/1000 match hours (CI 1.8 to 6.4) is lower compared with Ulster 6.01/1000 match hours (CI not reported).^11^ The Irish Rugby Football Union (IRFU) provides graduated return to play (GRTP) guidelines and requests injury reporting of all suspected/confirmed concussions which is independent of these studies. This study can add to the current knowledge base on concussion reporting and is the first report of concussion rates in the Leinster schoolboys cohort.

The tackle continues to be the most injurious event reported across schoolboy, amateur and professional studies.^11 12 29 32 33^ Tackle height and tackle proficiency have received much focus since the completion of the SCRUm study. World Rugby recently approved global trials of lower tackle height (below sternum) in community rugby.^34^ This trial is informed by professional studies which showed below shoulder level contact and bent-at-waist tackler had lower injury risk.^35^ The IRFU has adopted the new tackle height trial and provides an online education module and webinars to support uptake of this law.^36^

Match injuries had a median time loss of 22 days (95% CI: 17 to 28) compared with 24 (SD=20) days in Ulster schoolboys,^11^ and 13 to 29 days in two English schoolboy studies.^32 33^ Training injuries had a median time loss of 14 days (95% CI: 5 to 41) compared with 9 days in English schoolboys.^23^ The median time loss for concussion was within the mandated 23-day GRTP guidelines for adolescent concussion management.^37^

Training practices have not previously been reported in an Irish schoolboy cohort. This study estimated training mean frequency of three (SD=1.4) sessions weekly, per school. Recent guidance on training frequency in adolescent and adult rugby players considers the contextual factors of preparing for competition and injury reduction in rugby, with 2–3 sessions of contact load and tackle training suggested per week.^38 39^ Similarly, World Rugby’s Activate movement control exercise programme for injury prevention requires participation three times weekly.^25 40^ The Engage programme developed by the IRFU is designed to enhance rugby performance and reduce injury risk.^41^ This 15 min warm-up programme suitable for all levels of the game, including schools level, includes dynamic movement, muscle activity exercise and rugby-specific activities. This holds promise as an injury prevention strategy but needs evaluation to determine effectiveness. This study helps to inform frequency of rugby training in schoolboy rugby which may inform the potential uptake of injury prevention strategies such as Activate and Engage programmes.

Variation in the proportion of training types was evident across Leinster schools. While an English schoolboy study reported similar training session types, proportions differed.^23^ Training sessions can incorporate multiple types of training within a single session, which can pose challenges in delineating different types of training. The English study (n=222, 7 schools) reported 15 877 hours of training exposure over two seasons which is lower compared with our study.^23^ Contextual factors in these cohorts, including participation in rugby training, differing squad sizes and training exposures may be contributing to the variance in injury incidences, but these factors remain difficult to capture to allow prediction of injury risk. Therefore, the training data serve as a description of training practices in this cohort.

### Study limitations

The SCRUm study had a 20-week data collection period, which missed up to 8 weeks of the early season, and up to 2 weeks at the end of season lost due to COVID-19 pandemic restrictions. A full season of data collection is recommended.^14^ Despite early engagement with schools, a lengthy recruitment period (September to November 2019) contributed to the later start date of data collection, due to players’ academic commitments and difficulty accessing school personnel.

Players’ reporting of training load (RPE) was low, with approximately 88.5% of missing RPE data. Low compliance in self-reported measures of training load has previously been reported.^42^ Players were permitted to record 7 days post-activity to reduce the burden of data collection. Recall error increases with time and the maximum acceptable time period was used.^18^ Researchers aimed to engage with players on a weekly basis to support data collection, but this was not consistently feasible with competing academic commitments. With improved data collection, making the calculation of ACWR possible, the relationship between ACWR and injury risk could be explored, as this has not yet been reported in this cohort and was not possible in this study.

Exposure data were estimated at a team rather than individual level, which may result in over or underestimation of training exposure and subsequently impact injury incidence calculation. To improve accuracy of exposure calculation, this requires availability of coaching and support staff to record training frequency, duration and number of players participating in each training session.

Most injury data collectors were non-medical personnel in this study, in line with results of a cross-sectional survey of 93 rugby-playing Irish schools, where 86% of injury data collectors were non-medical personnel.^43^ The potential for under or over-reporting of injuries by non-medical personnel has previously been acknowledged.^11 44^ Medical verification of injuries was not feasible in this cohort, but use of non-medical personnel in injury surveillance is deemed acceptable at a community level^45 46^ for community rugby injury surveillance to engage non-medical personnel to contribute to improving player welfare.

### Future recommendations

Future community injury surveillance may benefit from categorising where diagnoses are medically verified to improve incidence calculation. Further surveillance of training practices of players may inform the contribution of training types to match preparedness and skill proficiency, and analysis of association with injury risk. Long-term injury surveillance in this cohort and expansion to the female game will further inform injury trends and potential injury risk factors. Coach and player advisory groups could inform and consolidate current training practices, barriers and motivators to recording training load and implementing future injury prevention strategies.

## Conclusion

The SCRUm study is the first to report injury incidence and training practices in Leinster senior schoolboy rugby players. Match and training injury incidences were lower compared with similar Irish studies, but within the range reported by a recent meta-analysis of similar cohorts. Injury site, mechanism and severity were comparable to similar Irish and international studies. Tackle continues to incur the highest proportion of injuries. Training practices differed across the schools, with similar training session types but varying proportions of training types undertaken. Ongoing research in the adolescent game is necessary to inform injury trends and explore specific injury risk factors for this cohort.

## Supplementary material

10.1136/bmjsem-2025-002535 online supplemental file 1

## Data Availability

All data relevant to the study are included in the article or uploaded as supplementary information.
