# Supplementary material for: Training load and injury surveillance in Leinster SChoolboy RUgby players: the SCRUm cohort study
Source: BMJ Open Sport Exerc Med. 2025 Aug 28;11(3):e002535. doi: 10.1136/bmjsem-2025-002535 (PMC12410643; doi:10.1136/bmjsem-2025-002535)

**TITLE:**

**Training load and injury surveillance in Leinster SChoolboy RUgby players: the SCRUm cohort study**

Sarah J. Murphy^1^, Louise Keating^1^, Ronan Conroy^2^, Jennifer Murphy^1^, Chloe Leddy^1^, David Clancy^1^, Chris M. Bleakley^3^, John Quinlan^4^, Cliff Beirne^4^, Helen P French^1^

^1^School of Physiotherapy, Royal College of Surgeons in Ireland (RCSI) University of Medicine and Health Sciences, Dublin, Ireland

^2^School of Population Health, Royal College of Surgeons in Ireland (RCSI) University of Medicine and Health Sciences, Dublin, Ireland

^3^School of Health Sciences, Ulster University, Coleraine, United Kingdom

^4^Irish Rugby Football Union Charitable Trust, Dublin, Ireland

Corresponding author: Sarah J. Murphy, sarahjmurphy@rcsi.com

# Supplemental Material

Supplemental Material 1 Mechanism of injury, by activity, region, incidence and severity

| **Match Injuries** | | | | **Training Injuries** | | | |
| --- | --- | --- | --- | --- | --- | --- | --- |
|  |  | **Incidence** | **Median**  **time loss** |  |  | **Incidence** | **Median**  **time loss** |
| **Mechanism** | **n** | **per 1000 Match hours** (95% CI) | **Days** (95% CI) | **Mechanism** | **n** | **per 1000 Training hours** (95% CI) | **Days** (95% CI) |
| Region |  |  |  | Region |  |  |  |
| Total | 61 | 19.9 (17.2-28.9) | 22 (17-28) | Total | 23 | 0.7 (0.5-1.1) | 14 (5-41.5) |
| **Tackling** | **21** | **6.9 (4.8-11.8)** | **21 (14-28)** | **Tackling** | **4** | **0.1 (0.0-0.4)** | **18 (6-43*)** |
| Shoulder | 10 | 3.3 (1.8-6.8) | 21.5 (4.8-62) | Ankle | 1 | 0 (0.0-0.2) | 43 (43-43*) |
| Head | 6 | 2 (0.8-4.8) | 16 (3.2-46.5) | Head | 1 | 0 (0.0-0.2) | 21 (21-21*) |
| Wrist/Hand | 2 | 0.7 (0.1-2.7) | 32 (19-45*) | Shoulder | 1 | 0 (0.0-0.2) | 6 (6-6*) |
| Chest | 1 | 0.3 (0.0-2.1) | 28 (28-28*) | Wrist/Hand | 1 | 0 (0.0-0.2) | 15 (15-15*) |
| Elbow | 1 | 0.3 (0.0-2.1) | NR |  |  |  |  |
| Hip/Groin | 1 | 0.3 (0.0-2.1) | NR |  |  |  |  |
| **Tackled** | **16** | **5.2 (3.4-9.6)** | **27 (12-42)** | **Tackled** | **0** |  |  |
| Head | 5 | 1.6 (0.6-4.3) | 29.5 (2-41*) |  |  |  |  |
| Shoulder | 5 | 1.6 (0.6-4.3) | 21 (12-47*) |  |  |  |  |
| Ankle | 1 | 0.3 (0.0-2.1) | 36 (36-36*) |  |  |  |  |
| Knee | 1 | 0.3 (0.0-2.1) | 79 (79-79*) |  |  |  |  |
| Lumbar Spine | 1 | 0.3 (0.0-2.1) | 56 (56-56*) |  |  |  |  |
| Thigh | 1 | 0.3 (0.0-2.1) | 15 (15-15*) |  |  |  |  |
| Trunk/Abdo | 1 | 0.3 (0.0-2.1) | 2 (2-2*) |  |  |  |  |
| Wrist/Hand | 1 | 0.3 (0.0-2.1) | 12 (12-12*) |  |  |  |  |
| **Collision** | **11** | **3.6 (2.0-7.3)** | **17.5 (5-27)** | **Collision** | **4** | **0.1 (0.0-0.4)** | **7 (7-7*)** |
| Head | 6 | 2 (0.8-4.8) | 17 (4-40*) | Wrist/Hand | 2 | 0.1 (0.0-0.3) | NR |
| Shoulder | 2 | 0.7 (0.1-2.7) | 23 (18-28*) | Ankle | 1 | 0 (0.0-0.2) | NR |
| Wrist/Hand | 2 | 0.7 (0.1-2.7) | 13 (4-22*) | Shoulder | 1 | 0 (0.0-0.2) | 7 (7-7*) |
| Chest | 1 | 0.3 (0.0-2.1) | 13 (13-13*) |  |  |  |  |
| **Running** | **5** | **1.6 (0.6-4.3)** | **36 (26-59*)** | **Running** | **4** | **0.1 (0.0-0.4)** | **17 (1-196*)** |
| Thigh | 2 | 0.7 (0-0.2) | 27 (26-28*) | Thigh | 2 | 0.1 (0.0-0.3) | 6.5 (1-12*) |
| Ankle | 1 | 0.3 (0.0-2.1) | 59 (59-59*) | Ankle | 1 | 0 (0.0-0.2) | 196 (196-196*) |
| Knee | 1 | 0.3 (0.0-2.1) | 44 (44-44*) | Hip/Groin | 1 | 0 (0.0-0.2) | 22 (22-22*) |
| Lower Leg | 1 | 0.3 (0.0-2.1) | NR |  |  |  |  |
| **Ruck** | **3** | **1 (0.2-3.2)** | **57.5 (52-63*)** | **Ruck** | **4** | **0.1 (0.0-0.4)** | **4 (3-48*)** |
| Head | 2 | 0.7 (0-0.2) | 52 (32-32*) | Shoulder | 2 | 0.1 (0.0-0.3) | 3.5 (3-4*) |
| Wrist/Hand | 1 | 0.3 (0.0-2.1) | 63 (63-63*) | Ankle | 1 | 0 (0.0-0.2) | 48 (48-48*) |
|  |  |  |  | Head | 1 | 0 (0.0-0.2) | NR |
| **Other** | **3** | **1 (0.2-3.2)** | **22 (8-32*)** | **Other** | **3** | **0.1 (0.0-0.3)** | **31 (4-58*)** |
| Chest | 1 | 0.3 (0.0-2.1) | 8 (8-8*) | Foot | 1 | 0 (0.0-0.2) | 4 (4-4*) |
| Head | 1 | 0.3 (0.0-2.1) | 32 (32-32*) | Knee | 1 | 0 (0.0-0.2) | NR |
| Knee | 1 | 0.3 (0.0-2.1) | 22 (22-22*) | Shoulder | 1 | 0 (0.0-0.2) | 58 (58-58*) |
| **Maul** | **1** | **0.3 (0.0-2.1)** |  | **Maul** | **1** | **0 (0.0-0.2)** |  |
| Knee | 1 | 0.3 (0.0-2.1) | NR | Neck | 1 | 0 (0.0-0.2) | NR |
| **Not known** | **1** | **0.3 (0.0-2.1)** |  | **Not known** | **1** | **0 (0.0-0.2)** |  |
| Ankle | 1 | 0.3 (0.0-2.1) | 27 (27-27*) | Lumbar Spine | 1 | 0 (0.0-0.2) | 13 (13-13*) |
| **Scrum** | **0** |  |  | **Scrum** | **1** | **0 (0.0-0.2)** |  |
|  |  |  |  | Shoulder | 1 | 0 (0.0-0.2) | NR |
| **Lineout** | **0** |  |  | **Lineout** | **1** | **0 (0.0-0.2)** |  |
|  |  |  |  | Ankle | 1 | 0 (0.0-0.2) | 40 (40-40*) |

* Lower (upper) confidence limit held at minimum (maximum) of sample; NR, not reported

Supplemental Material 2 Injury frequency, By playing position, By activity

| **Position Groups** | **Match injuries (n)** | **Training injuries (n)** | **Total** |
| --- | --- | --- | --- |
| **Forwards** | **39** | **8** | **47** |
| ***Front row*** | ***14*** | ***3*** | ***17*** |
| *1. Loosehead Prop* | *5* | *1* | *6* |
| *2. Hooker* | *5* | *2* | *7* |
| *3. Tighthead Prop* | *4* | *0* | *4* |
| ***Second row*** | ***7*** | ***4*** | ***11*** |
| *4. Left Lock* | *4* | *2* | *6* |
| *5. Right Lock* | *3* | *2* | *5* |
| ***Back row*** | ***18*** | ***1*** | ***19*** |
| *6. Blindside Flanker* | *4* | *1* | *5* |
| *7. Openside Flanker* | *10* | *0* | *10* |
| *8. Number 8* | *4* | *0* | *4* |
| **Backs** | **22** | **10** | **32** |
| ***Scrum half*** | ***2*** | ***1*** | ***3*** |
| ***Fly half*** | ***5*** | ***4*** | ***9*** |
| ***Centres*** | ***9*** | ***2*** | ***11*** |
| *12. Inside Centre* | *6* | *1* | *7* |
| *13. Outside Centre* | *3* | *1* | *4* |
| ***Wingers*** | ***4*** | ***1*** | ***5*** |
| *11. Left Wing* | *4* | *1* | *5* |
| *14. Right Wing* | *0* | *0* | *0* |
| ***Full backs*** | ***2*** | ***2*** | ***4*** |
| **Position unknown** | **0** | **5** | **5** |
| **Total** | **61** | **23** | **84** |

Supplemental Material 3 Training session types, by percentage, by school


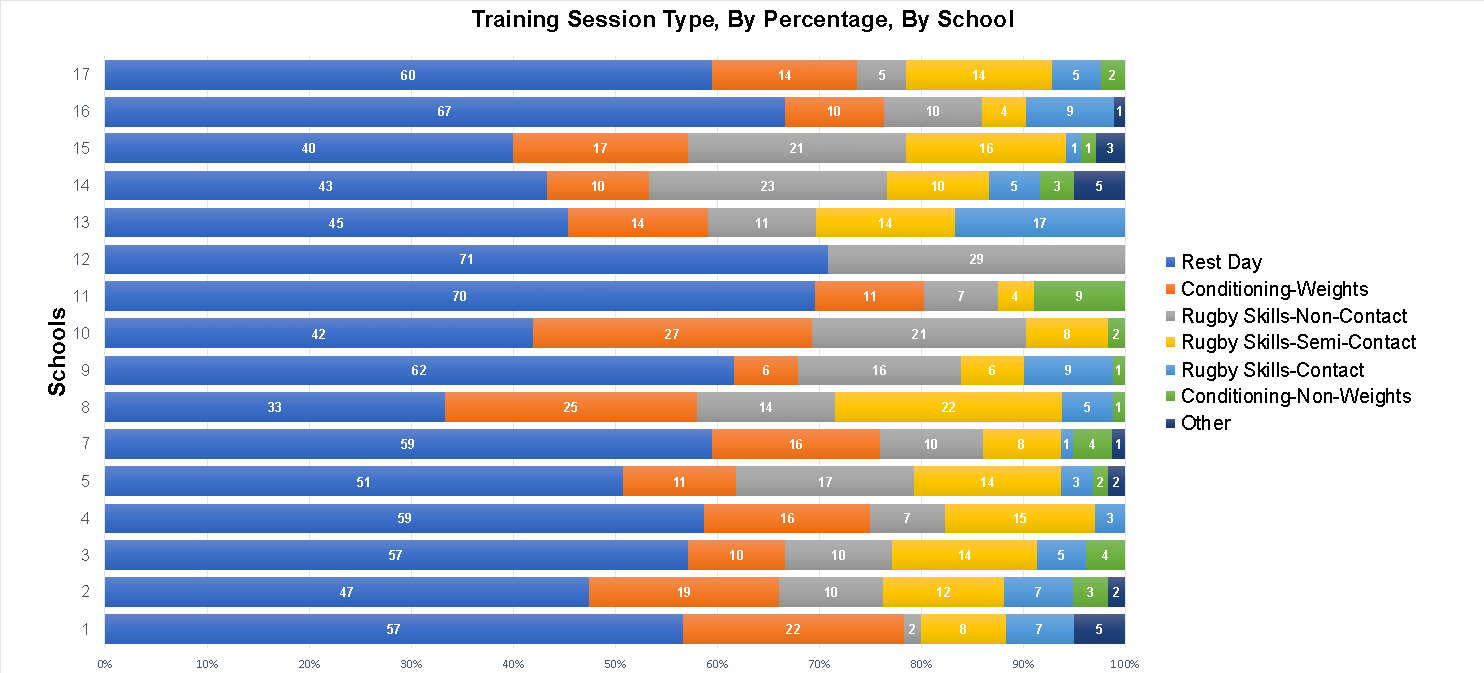

Supplement: online supplemental file 1 [file bmjsem-11-3-s001.docx]
